# Genito-Urinary and Syphilitic Diseases

**Published:** 1898-09

**Authors:** Byron H. Daggett

**Affiliations:** Buffalo, N. Y.


### GENITO-URINARY AND SYPHILITIC DISEASES.

CONDUCTED BY BYRON H. DAGGETT, M. D., Buffalo, N. Y.

### MOVABLE KIDNEY AND ITS TREATMENT.

MAX EINHORN (*New York Medical Record*, August 13, 1898,) says that floating kidney was known in the middle ages, but its clinical significance was not recognised until this century. Rayer, in 1836, first gave an accurate description of this condition.

In reference to treatment Rayer says: '' Experience has shown me that most commonly it is sufficient to support the abdomen by means of a properly applied bandage, in order to cause the disappearance of the pain, due to the movable right kidney, or at best to render it more endurable.'' Charles J. Horn, in the *Med. Times and Gazette* for January, 1858, said: '' This movable condition of the kidneys is not common, but is more so than is generally supposed and must present itself occasionally to most medical men engaged in a large practice. It is more common among women than men and occurs most often upon the right side. The habitual position of a movable kidney is lower than natural and the kidney may be felt below the costal cartilages, if the abdominal walls are thin and flaccid.

Hare gives the following directions for doing bimanual palpation: "Suppose the right side is to be examined; the patient being laid on the back, with a slight inclination toward the right side, the head and shoulders being raised and the legs somewhat elevated, the observer should stand or sit to the right of the couch, but somewhat facing the patient, and should place the fingers of the left hand on the postero-lumbar region, immediately under the last rib, at the same time gently pressing or pushing forward that part. The ends of the fingers of the right hand should then be placed in front, just below the costal cartilages and there also a slight pressure should be exerted. The lower end of the kidney will probably be felt between the two hands. The patient should next be told to take a deep inspiration and then to expire slowly; the observer should in the meanwhile keep his hands in the same position as before, but, just at the commencement of expiration, should press the fingers of the right hand rather sharply down toward the renal region. He will probably detect a much larger portion of the kidney between the hands than previously; it has been detruded downward by the action of the diaphragm. Sometimes the kidney may thus be retained between the hands, and now and then it may be detruded much lower into the abdomen, but it usually slips away from between the fingers, and is lost for a moment under the liver or in the proper renal region. In other cases, when the mobility is greater, if at the moment of commencing expiration (after a deep inspiration) the right hand be pressed edgewise along the margin of the costal cartilages, and sharply downward toward the renal region, the kidney will very probably glide or slip downward, so that the whole of it will be below the hand—below, that is, the level of the position where its inferior border is usually situated. In other instances, without this manipulation, the kidney is felt as a body so movable that it can be taken up, as it were, through the parieties, and be shifted or pushed to different parts of its own side of the abdomen, or even moved to the front of the spine. The degree of mobility, therefore, varies very much in different cases; it may amount to but a slight departure from the usual tolerably fixed condition of these organs, while, on the other hand, I have met with a case in which the right kidney could readily be moved upward and downward, over a space of between four and five inches and to a considerable extent transversely across the right half of the abdomen."

Hare claims that a well fitting elastic abdominal support gives the most efficient relief, providing that such support does not force the kidney above or below its normal position.

Kepler, of Berlin, in 1879, advised nephrectomy, but a death-rate of 27 per cent. resulted and objections were raised against such a severe and dangerous procedure. In 1881, E. Hahn, proposed fixation or nephrorrhaphy for the cure of movable kidney.

Edibohls perfected the technique of this operation and reported fifty operations. (*Amer. Jour. Med. Sciences*, March and April, 1893).

Landau, of Berlin, opposed any operative interference in this affection.

Glenard, by his masterly work, advanced our knowledge.

Glenard demonstrated that it was not the kidney alone that is displaced downward, but that other abdominal organs became looser and more movable and descended more or less ; that descending kidney was one of the concomitants of enteroptosis and falling of the stomach.

The study of enteroptosis enlarged our knowledge of floating kidney. "The surgical treatment of floating kidney has never, and with justice, been relegated to the background ; for, as this condition is only one of the concomitant appearances of enteroptosis, very little benefit is derived, as a matter of fact, from a nephrorrhaphy."

Dr. Einhorn reports observations of nearly 500 cases of movable kidney and summarises as follows : A floating kidney is a condition in which the kidney is more or less accessible to palpation. The kidneys are located in the posterior or upper part of the abdomen, partly within the bony walls of the thorax, at the sides of the transverse processes of the vertebræ, the inner margin being about 7.5 c. m. from the median line. They extend from the lower border of the eleventh dorsal vertebra to the lower border of the second lumbar vertebra ; they are inaccessible to palpation.

Glenard divides the different degrees of nephroptosis into four divisions. (1) The lower part of the kidney can be felt during inspiration ; (2) The greater part can be felt and grasped during inspiration ; (3) During deep expiration the upper border of the kidney can be reached ; (4) The entire kidney is accessible during deep expiration.

*Etiology.*—Nephroptosis occurs most frequently in women and most frequently upon the right side. Senator said that oue woman in 171 suffered from floating kidney. Glenard found this anomaly in one out of every four women. Lindner, one in every six women. According to Kuttner four out of 100 cases occurred in men. Hilbech never met with a palpable left kidney.

Einhorn, out of 1315 cases of digestive disturbances, found 126 floating kidneys, 772 were males with fourteen nephroptoses and 540 women presented 112 cases. One-third of the cases had gastroptosis. Of the 112 women with floating kidneys, five had ptosis of spleen. Enteroptosis occurs oftener with than without nephroptosis.

*Causes.*—The corset and tight waist bands are recognised causes. Landau and Sultzer allege that labor is responsible for this condition, due to the relaxation that follows child-birth. Rapid emaciation is regarded as a cause of renal displacements. Boequit states that congestion of the kidneys during mensturation is an etiological factor.

*Symptoms.*—(1) Weight and traction in the abdomen ; (2) Palpitation in the epigastrium, due to aortic pulsation ; (3) Symptoms are aggravated in the upright position and disappear in the horizontal. (4) Frequent urination, sometimes attended with burning sensation ; (5) Pain in sacral region after exertion ; (6) Discomfort, usually increased during menstruation and palliated in pregnancy. Most of the gastric and intestinal symptoms are due to concomitant abnormalities. Nephroptosis seldom cause gastric neuroses.

*Diagnosis.*—To recognise a floating kidney practice palpation as described above. Differentiation between a gall bladder, filled with calculi, a distended section of the intestine or any other abnormal condition is not often a difficult matter.

*Treatment.*—Rayer, Hare, Glenard and others recommend the abdominal bandage. There are surgeons who question the value of the bandage. Edibohls says it is a poor second to nephrorrhaphy. H. E. Lewis says that nonsurgical treatment has never more than a palliative effect. Extirpation, as proposed by Keppler is discarded. Nephrorrhaphy, as recommended by Hahn and practised by Edibohls, Senn and others is a recognised surgical procedure. Edibohls says the symptoms may be improved by dorsal posture, the Weir-Mitchell treatment, massage, electricity and abdominal bandages ; all these measures fail in the vast majority of cases, and even the improvement obtained is transient.

Edibohls also claims as a result of his experience that nephrorrhaphy properly performed, in appropriate cases, always improves and often permanently cures. Lewis is a vigorous advocate of operative treatment. Landau opposes all operative treatment. Huber says : " In reference to the therapeutics of enteroptosis, I would emphasise that, up to the present time, I have never been induced, owing to the severity of the symptoms or inefficacy of the adopted nonoperative treatment, to refer the patients to the surgeon for the performance of nephrorrhaphy, the most radical treatment which at present still appears admissible. This much can be said, at any rate : that an intelligent application of bandages in connection with an appropriate dietary and medicinal treatment of the gastric symptoms, will, as a rule, be adequate.

Einhorn advises medical treatment and gives the following reasons for his faith : (1) Dietetic, mechanical treatment produces very favorable results, providing that the gastric and intestinal symptoms are relieved by modern methods of treatment and an appropriate bandage is applied ; (2) Many cases of floating kidney manifest no symptoms. We incidentally find floating kidney when examining patients suffering from disturbances of the abdominal organs. Digestive disturbances seldom depend upon movable kidney, hence operation to cure the kidney would not cure the gastro-intestinal disturbance. Movable kidney is only one of the manifestations of enteroptosis and anchorage of the kidney would not cure the ptosis of the other organs ; (3) Nephrorrhaphy does not relieve the symptoms in the presence of nephroptosis any better than does rational medical treatment. According to Sulzer, the results of operation are unsatisfactory in one third of the cases. In addition to this there is a mortality of 2 per cent. and concomitants of severe and extensive operative procedure Floating kidney is generally associated with ptosis of other abdominal organs, hence nephrorrhaphy does not relieve the gastric and intestinal symptoms. Nephrorrhaphy is rarely justifiable and only when other treatment has failed. The vast majority of patients suffering from floating kidney may be relieved without any surgical intervention.

---

Duration of Immunity after Vaccination.—According to Jasiewicz, the immunity from vaccination lasts a much shorter time in infants than is generally supposed. A study of the statistics of the subject showed a proportion of 7.35 per cent. out of twenty-three children under six years of age in whom vaccination was successfully performed. The author, therefore, recommends more frequent re-vaccination in childhood and expresses the opinion that children are also protected from other infectious diseases thereby.—*Jour. de Clin. et Thér de l'Enfants.*—*Universal Med. Jour.*

